# Cellular senescence in Alzheimer’s disease: from physiology to pathology

**DOI:** 10.1186/s40035-024-00447-4

**Published:** 2024-11-20

**Authors:** Jing Zhu, Chongyun Wu, Luodan Yang

**Affiliations:** 1 Department of Pulmonary and Critical Care Medicine, The Central Hospital of Wuhan, Tongji Medical College, Huazhong University of Science and Technology, Wuhan, 430014 Hubei China; 2 Laboratory of Exercise and Neurobiology, School of Physical Education and Sports Science, South China Normal University, Guangzhou, 510006 Guangdong China

**Keywords:** Cellular senescence, Alzheimer’s disease, Senolytics, Senomorphics

## Abstract

Alzheimer’s disease (AD) is one of the most common neurodegenerative disorders, characterized by the accumulation of Aβ and abnormal tau hyperphosphorylation. Despite substantial efforts in development of drugs targeting Aβ and tau pathologies, effective therapeutic strategies for AD remain elusive. Recent attention has been paid to the significant role of cellular senescence in AD progression. Mounting evidence suggests that interventions targeting cellular senescence hold promise in improving cognitive function and ameliorating hallmark pathologies in AD. This narrative review provides a comprehensive summary and discussion of the physiological roles, characteristics, biomarkers, and commonly employed in vivo and in vitro models of cellular senescence, with a particular focus on various cell types in the brain, including astrocytes, microglia, oligodendrocyte precursor cells, neurons, and endothelial cells. The review further delves into factors influencing cellular senescence in AD and emphasizes the significance of targeting cellular senescence as a promising approach for AD treatment, which includes the utilization of senolytics and senomorphics.

## Background

Cellular senescence was first discovered almost 60 years ago in multiple strains of fibroblasts cultured in vitro [1, 2]. It was initially identified as a stable exit from the cell cycle caused by limited proliferation of normal human fibroblasts [1, 2]. Currently, cellular senescence is defined as an irreversible cell cycle arrest in response to either intrinsic or extrinsic insults, including telomere shortening and aberrant oncogenic activation (e.g., overexpression of HrasV12), mitochondrial dysfunction, oxidative and genotoxic stress, irradiation, and chemotherapeutic agents [3]. Therefore, senescence is also considered a defense mechanism against unnecessary cellular damage [4]. Cellular senescence is closely associated with aging [5]. As previously reported, hallmarks of aging are divided into three main categories: (1) primary hallmarks, causes of age-related cellular damage; (2) antagonistic responses in response to cellular damage; and (3) integrative hallmarks, consequences of the responses and culprits of the clinical phenotype [5]. Senescence belongs to the antagonistic class [5]. However, increasing evidence shows that the accumulation of senescent cells has deleterious effects on the tissue microenvironment by secreting pro-inflammatory and matrix-degrading molecules (known as senescence-associated secretory phenotype, SASP) [6–8]. SASP contributes to the pathological progression of aging-related neurodegenerative disease [6–8].

Aging is the most significant risk factor for AD [9]. As the most common form of dementia among older people [6], AD-associated cellular senescence has recently attracted increasing attention [6, 7, 10]. AD treatment strategies in the past 30 years were mainly focused on the pathological hallmarks, i.e., extracellular beta-amyloid (Aβ) accumulation and intraneuronal abnormal tau hyperphosphorylation [11]. However, there are currently no effective pharmacotherapeutic approaches for AD prevention and treatment [12]. Recent studies suggest that astrocytes, microglia, and oligodendrocyte progenitor cells (OPCs) exhibit a senescence phenotype in the Aβ plaque environment [7, 13, 14]. These studies provide a promising new avenue for studying senolytic strategies in AD.

In this narrative review, we provide a comprehensive overview and discussion of the physiological roles, characteristics, biomarkers, and models of cellular senescence, with a specific focus on various brain cell types, including astrocytes, microglia, OPCs, neurons, and endothelial cells. We synthesize existing knowledge from both primary research and relevant reviews to explore the factors influencing cellular senescence in AD and highlight the therapeutic potential of targeting cellular senescence, including the use of senolytics and senomorphics.

## Understanding cellular senescence: definitions, classifications, and key characteristics

### Definition and classification of cellular senescence

Cellular senescence is classified into replicative senescence and stress-induced premature senescence (SIPS) [15]. It is characterized by a stable cell cycle arrest triggered by developmental signals or by intrinsic and extrinsic stimuli [15]. Replicative senescence refers to the process of normal, nonmalignant cells undergoing cell cycle arrest after a limited number of divisions [16]. Key factors leading to replicative senescence include sustained proliferation, telomerase deficiency, and telomere shortening [15]. Most primary cells have low telomerase levels, leading to cell growth arrest after several vigorous generations due to telomere shortening and chromosomal fusions [17]. Supporting this concept, some studies showed that telomere shortening leads to p53 activation, a key mediator of cellular senescence, which further promotes senescence [18, 19]. External and internal stresses also induce cellular senescence, which is termed SIPS and occurs independently of telomerase length [20]. Chemical or physical stimuli-induced stress, including oxidative stress, inflammation, mitochondrial dysfunction, suboptimal culture conditions, and various genotoxic stressors, lead to SIPS [21]. For instance, superoxide radicals, ultraviolet radiation, and hypoxia can induce cellular senescence in cultured cells [22–24]. Replicative senescence and SIPS share characteristics such as growth arrest, telomere shortening, DNA damage, p53 activation from DNA damage accumulation, and altered expression of proteins involved in cell-cycle regulation [25, 26]. Thus, SIPS is frequently used as a model to study normal aging [25].

### Key features of cellular senescence

#### Growth arrest

As mentioned previously, one of the hallmarks of cellular senescence is the irreversible cell cycle arrest [3]. Senescent cells can not initiate DNA replication despite adequate growth conditions and do not respond to growth factors or mitogenic stimuli [27]. The growth arrest is induced by the expression of cyclin-dependent kinase (CDK) inhibitors described below in detail. Like the terminally differentiated cells, the senescent cells are irreversibly withdrawn from the cell cycle. However, unlike differentiated cells, senescent cells are not specialized effector cells and have no specific physiological function [28]. Growth arrest is a potent tumor-suppressive mechanism, but it is not conducive to neuronal survival and damage repair [29].

#### Apoptosis resistance

Factors that determine cell fate choice between apoptosis and senescence remain unclear. However, cell type, intensity and nature of the stress/stimuli/damage, and changes of cellular apoptosis-related proteins may determine the direction of cell fate [27]. In contrast to apoptotic cells, senescent cells exhibit anti-apoptotic properties [30]. Senescent cells display significantly increased levels of anti-apoptotic proteins BCL-W and BCL-XL, which result in apoptosis resistance [31]. The anti-apoptotic properties induce an accumulation of senescent cells without function, contributing to normal aging and promoting the development of age-related disorders [32].

#### SASP

Although senescent cells are growth-arrested, they are still in a metabolically active state and display a hyper-secretory phenotype termed SASP [8]. SASP is a fundamental feature of cellular senescence [33]. In AD, SASP occurs in different cell lines and leads to secretion of various pro-inflammatory cytokines, including IL-6, IL-8, TNF-α, TGF-β, and IL-1β [7]. The excessive release of pro-inflammatory cytokines exacerbates pathological changes in AD, including Aβ accumulation, neurofibrillary tangles, neuronal loss, and neuronal degeneration [7, 34].

#### DNA damage response (DDR)

DDR is initiated by DNA damage or telomere shortening. In addition to arresting cell-cycle progression, cells respond to DNA damage by attempting to repair DNA damage via DDR pathways in aging. Progressive telomere shortening also triggers a DDR, and the markers of DDR are localized to the telomeres [35]. The DDR includes formation of DNA damage foci at either uncapped telomeres or persistent DNA strand breaks, which contribute to cellular senescence [36]. Indeed, cellular senescence can be considered a permanent DNA-damage response state [37].

### Biomarkers of cellular senescence

#### p16^INK4a^ in cellular senescence

The p16^*INK4a*^ gene, also called *CDKN2a* (cyclin-dependent kinase inhibitor 2A), is located on chromosome 4 in mice and chromosome 9, band p21.3 in humans [38]. The p16^*INK4a*^ gene encodes two proteins, p16^INK4a^ and p14^arf^ [39]. The p16^*INK4a*^ gene, first identified as *MTS1* (multi-tumor suppressor 1) in1994, is inactivated or mutated in nearly 50% of human cancers [40], which suggests an essential role of p16^INK4a^ in the cell cycle [41]. The cell cycle has four stages called G1, S, G2, and M phases [42]. Cell cycle checkpoints regulate progression through these four phases [43]. The G1 checkpoint controls the G1-to-S transition, and p16^INK4a^ is a critical component of the G1 checkpoint [39, 44]. p16^INK4a^ binds to CDK4 and CDK6, inhibits their activity, and prevents retinoblastoma (Rb) tumor suppressor protein phosphorylation. The dephosphorylated status of Rb physically associates with E2Fs (E2F1-E2F3) and blocks their transactivation domain [45]. Moreover, the interaction between the E2F and Rb transactivation domain inhibits the expression of E2F1 target genes that are crucial for G1/S transition [45, 46]. Therefore, the increased level of p16^INK4a^ can induce G1 cell cycle arrest in aged and stressed tissues [47]. Clearance of p16^INK4a^-positive senescent cells protects against AD and other aging-associated disorders [7, 48, 49]. Now, p16^INK4a^ has been regarded as a marker of aging and cellular senescence, although the currently available antibodies poorly detect p16^INK4a^ in mice [50].

#### p21^CIP1/WAF1/SDI1^ in cellular senescence

p21^CIP1/WAF1/SDI1^ is another potent CDK inhibitor [51]. The p21-encoding gene was initially found as a target of p53 to mediate its role as a tumor suppressor [52]. p21 interacts with CDK2-associated complexes and inhibits the cell cycle from entering the S phase [51]. Therefore, as a downstream target of p53, p21^CIP1/WAF1/SDI1^ is activated to trigger cell cycle arrest and contributes to cell cycle arrest, apoptosis, and differentiation [51]. Consistently, p21^CIP1/WAF1/SDI1^ is closely associated with stress or danger cell responses in multiple tissues [53]. For example, p21 triggers the cell cycle growth arrest in the lung to modulate alveolar inflammation and destruction [53]. As a sensor of cellular stress, p21^CIP1/WAF1/SDI1^ in cellular senescence provides a logical explanation of how p21 is involved in arrested tissue repair but contributes to tumor suppression.

#### Senescence-associated beta-galactosidase (SA-β-gal) in cellular senescence

SA-β-gal is the most extensively utilized biomarker for cellular senescence [54]. Increased SA-β-gal activity can be detected as cells enter the senescent state [55]. A quantitative assay of SA-β-gal activity found that the normalized β-gal activity in senescent cells is two folds that in pre-senescent cells [56]. Therefore, assays measuring SA-β-gal activity offer approaches to detect cellular senescence. Several substrates for SA-β-gal are currently widely used in the assays, including 5-bromo-4-chloro-3-indolyl-β-D-galactopyranoside (known as x-gal), C_12_FDG, and CellEvent senescence green reagent [57–59]. The x-gal and C_12_FDG can be converted into a blue precipitate by SA-β-gal and used as an indicator for cellular senescence [58, 59].

#### Lipofuscin in cellular senescence

Lipofuscin is a nondegradable aggregate of oxidation products of covalently cross-linked proteins, lipids, and metals within lysosomes [60]. Lipofuscin accumulates with age and is considered a hallmark of senescent cells [61]. Lipofuscin is an autofluorescent material that can be detected using fluorescence microscopy [60]. Besides, Sudan Black B and the biotinylated chemical compound derived from Sudan Black B have been described as a method of detecting lipofuscin based on lipid detection [62].

## Cellular senescence in AD: contributing factors and cell types affected

### Factors contributing to cellular senescence in AD

**Fig. 1:**
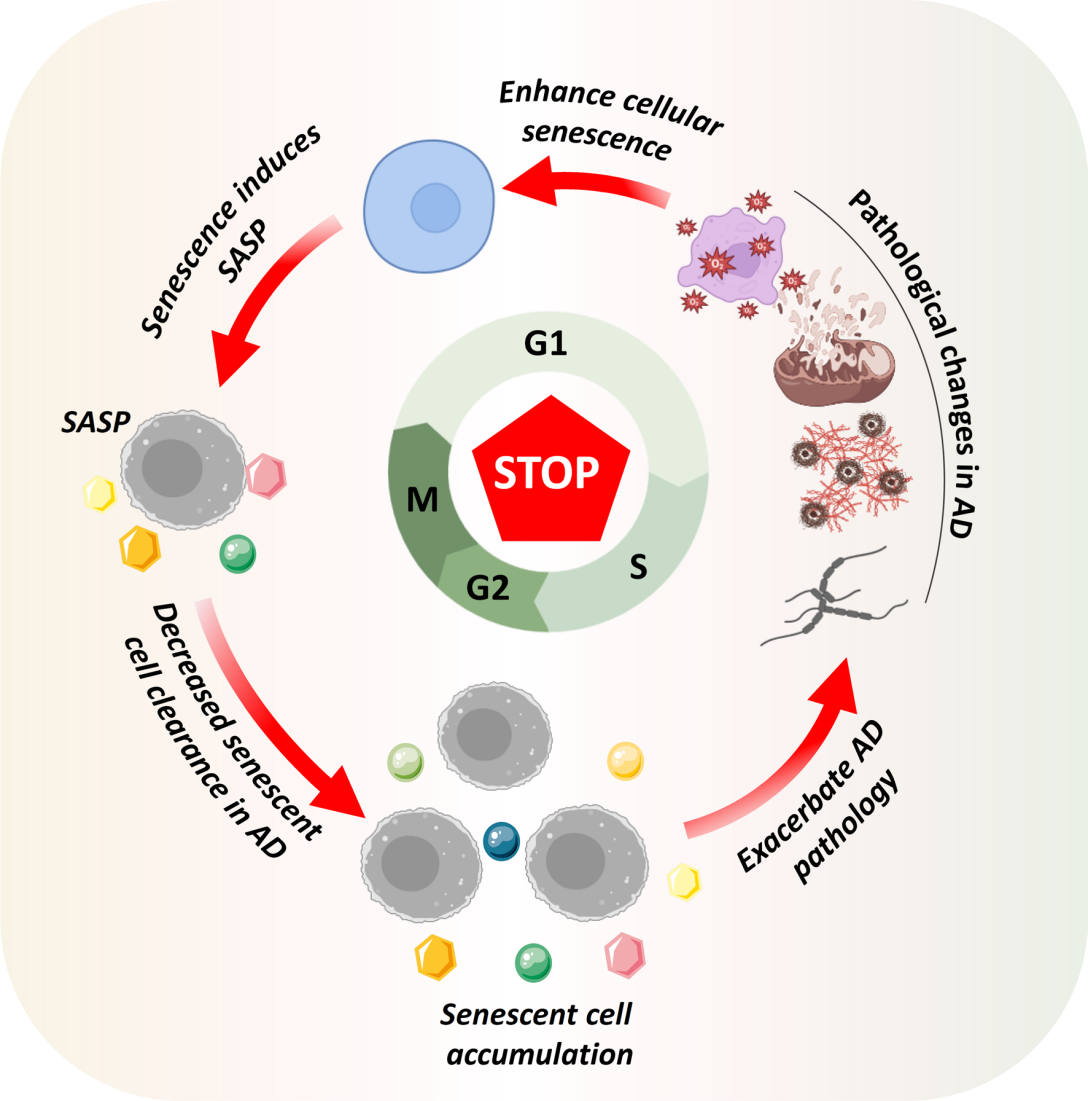
Pathological vicious cycle of cellular senescence in AD. Under pathological conditions of AD, cellular senescence induces SASP, leading to accumulation of senescent cells. This process is accompanied by excessive release of reactive oxygen species and pro-inflammatory factors, exacerbating mitochondrial dysfunction, oxidative stress, and inflammatory responses. Moreover, it accelerates Aβ deposition and excessive phosphorylation of tau, forming a vicious cycle between cellular senescence and AD pathology. SASP, senescence-associated secretory phenotype

Under pathological conditions of AD, cellular senescence induces the formation of SASP, leading to the accumulation of senescent cells. This process is accompanied by excessive release of reactive oxygen species (ROS) and pro-inflammatory factors, exacerbating mitochondrial dysfunction, oxidative stress, and inflammatory responses. Moreover, it accelerates amyloid-beta (Aβ) deposition and triggers excessive phosphorylation of tau, forming a vicious cycle between cellular senescence and AD pathology (Fig. 1). In addition, altered proteostasis, DNA damage and repair, telomerase deficiency, and telomere shortening also contribute to cellular senescence in AD [63–65]. The following paragraphs review and discuss factors influencing cellular senescence in AD.

#### Mitochondrial dysfunction

Mitochondrial dysfunction is a hallmark in neurodegenerative diseases and brain injuries [66, 67]. Disrupted mitochondrial structure, abnormal function, and impaired biogenesis have been observed in AD [66, 67]. Specifically, an imbalance between mitochondrial fusion and fission leads to excessive fragmentation, evidenced by decreased fusion-associated proteins (e.g., MFN1, MFN2, OPA1) and increased fission-associated proteins (e.g., FIS1, MFF, MIEF, DRP1) in the cortex and hippocampus [67, 68]. Additionally, defects of oxidative phosphorylation caused by impairment of mitochondrial enzymes such as Complexes II and IV, contribute to reduced ATP production [67, 68]. Impaired mitochondrial trafficking and defective mitophagy have also been observed in AD [69, 70].

Mitochondrial dysfunction is closely linked to cellular senescence [71]. Senescent cells exhibit reduced mitochondrial membrane potential and defects in respiratory chain complexes [71]. Deficiency of complex I assembly factors alone can induce cell senescence [72]. Indeed, mitochondrial DNA is essential for electron transport and mitochondrial function [73]. Mitochondrial genomic instability induces cellular senescence when mitochondrial DNA mutations are accompanied by increased ROS [73]. Additionally, mitochondrial content is associated with cellular senescence [71, 74]. Both in vivo and in vitro studies have observed elevated mitochondrial mass in senescence, possibly as a compensatory mechanism for mitochondrial dysfunction [75, 76]. Consistent with this, reducing mitochondrial mass significantly inhibits cellular senescence, suggesting that targeting mitochondria may help alleviate senescence [74]. Mitochondrial Ca^2+^ overload also mediates the essential role of mitochondria in cellular senescence [77]. Increasing evidence shows that mitochondrial Ca^2+^ overload contributes to AD pathology [77]. The mitochondrial Ca^2+^ overload causes decreased membrane potential and excessive production of ROS [71, 77]. Subsequently, the excessively released ROS causes DNA damage and telomere shortening, activating the p53 pathway and cellular senescence [64]. These findings suggest that mitochondrial dysfunction is a potential intervention target for alleviating cellular senescence in AD.

#### Oxidative stress

As mentioned previously, mitochondrial dysfunction-induced oxidative stress causes genomic DNA damage and activates cellular senescence-associated pathways [64]. Therefore, suppressing oxidative stress may be a way to inhibit cellular senescence [78]. According to previous studies, anti-oxidative substances and approaches potentially improve age-related neurodegenerative diseases by targeting cellular senescence [34, 66, 79]. For instance, curcumin, a natural phenol with antioxidant activities, exhibits therapeutic potential for AD treatment [80]. An in vitro study showed that curcumin significantly ameliorates hydrogen peroxide-induced endothelial cellular senescence [81]. Nicotinamide, a vitamin B3 derivative, significantly alleviates the replicative senescence of human fibroblasts by reducing ROS production [82]. Photobiomodulation, a non-invasive low-level laser therapy, directly improves mitochondrial function by promoting cytochrome *c* oxidase activity, which reduces electron leakage, thereby decreasing oxidative stress and mitigating the senescence of bone marrow mesenchymal stem cells [79]. Further, a previous study found that cellular ROS in neurons could induce cellular senescence by activating the IL-6/STAT3 pathway [83], and that inhibiting ROS production or the IL-6/STAT3 pathway alleviated cellular senescence [83]. These findings suggest that oxidative stress or its downstream targets may contribute to cellular senescence.

#### Neuroinflammation

AD is also accompanied by a robust inflammatory response characterized by excessive release of pro-inflammatory factors and overactivation of glial cells [66]. The neuroinflammatory response is triggered by the accumulation of Aβ, tau, and damage-associated molecular patterns (etc., mitochondrial constituents) [66]. At the early stage, glial cells, including astrocytes and microglia, are activated in response to the pathological changes [66, 84]. During this stage, they are involved in the clearance of pathological molecules through microglial phagocytosis and glymphatic clearance pathway [85, 86]. However, as the disease progresses, microglia and astrocytes fail to remove pathological molecules [66, 84], and chronically activated microglia and astrocytes excessively release pro-inflammatory cytokines and ROS, causing detrimental effects [66]. Indeed, the chronic activation of glial cells and excessive release of pro-inflammatory cytokines exacerbate mitochondrial dysfunction, oxidative stress, and accumulation of pathological molecules, forming a vicious cycle and contributing to cellular senescence [66]. A previous study observed decreased nicotinamide adenine dinucleotide (NAD^+^) levels and an increased neuroinflammatory response in AD mouse brains [87]. Exogenous NAD^+^ supplementation alleviates microglial and astrocytic activation and reduces pro-inflammatory cytokine release [87]. Interestingly, the decreased neuroinflammation is accompanied by attenuated senescence of brain cells, including microglia, astrocytes, neurons, and OPCs [87]. In fact, the pro-inflammatory secretory phenotype SASP, the hallmark of senescent cells, releases significantly increased pro-inflammatory cytokines, exacerbating pathological changes and cellular senescence, which highlights the intimate association between inflammation and cellular senescence [15].

#### Altered proteostasis

Proteostasis or protein homeostasis refers to appropriate protein synthesis, folding, post-translational modification, trafficking, and degradation [63]. Altered proteostasis is a common feature of several neurodegenerative diseases and has been extensively studied in AD [88–90]. Defective protein synthesis caused by dysfunctional mRNA translation control has been discovered as an early event and a trigger or a mediator of AD pathogenesis [91]. In addition, alterations of mechanistic target of rapamycin complex 1 (mTORC1) signaling can cause changes in proteostasis in AD [92, 93]. As part of mTORC1, the mechanistic target of rapamycin (mTOR) is a central nutrient-sensitive regulator and regulates cellular proliferation, metabolism, and protein synthesis [93]. mTOR exists in 2 complexes, mTORC1 and mTORC2, with different cellular functions [94]. mTORC1 plays a major role in protein synthesis and degradation [93]. mTORC1 is overactivated in AD and contributes to AD progression [95]. For example, although protein translation inhibition is observed in AD, overactivation of mTORC1 causes detrimental excessive synaptic protein synthesis [96]. Moreover, elevated mTORC1 activity suppresses autophagy in AD, consistent with the impaired autophagy and Aβ clearance in AD patients and animal models [95, 97].

Recent evidence indicates that altered proteostasis and cellular senescence share several pathogenic mechanisms, and cellular senescence works as an adaptive stress response to altered proteostasis [63]. In a previous study, prolonged neuronal culture displayed cellular senescence and altered proteostasis [63]. However, attenuation of the altered proteostasis with a mTOR antagonist alleviated the proteotoxic stress and reduced cellular senescence, suggesting that altered proteostasis contributes to cellular senescence [63]. Indeed, altered proteostasis is one of the typical features of cellular senescence [98]. A change of cellular state from normal functioning to the senescent state needs the participation of proteome alteration [98]. Furthermore, it should be noted that although stress sensing is remarkably promoted in human senescent cells, the stress adaptation at the gene transcription level is reduced in these cells because of the altered proteostasis [99].

#### DNA damage and repair

Emerging evidence supports the essential role of DNA damage and repair in healthy aging and various neurodegenerative diseases, including AD and multiple sclerosis [100, 101]. DNA repair maintains genomic stability during healthy aging and alleviates DNA damage [102]. In AD, increased DNA damage and dysfunctional DNA repair further facilitate AD pathogenesis [15, 103]. Increasing evidence supports that DNA damage is a crucial pathological cause of and an early event in AD [15, 104, 105]. The excessive release of ROS causes DNA–protein and DNA-DNA cross-links, formation of modified bases and DNA adducts, and double-stranded DNA breaks [105]. In response to DNA damage, DDR is initiated, which involves a series of events including DNA damage detection (damage sensors), repair, and cellular fate decision [106]. In aging, DNA damage and the activation of the DDR can influence cell fate decisions, leading to outcomes such as cell death or cellular senescence [107]. In AD, different cellular stressors (e.g., oxidative stress, DNA damage, and mitochondrial stress) combined with proteinopathy lead to DNA damage and a DDR cascade that causes senescence [108]. DNA damage contributes to the activation of DDR machinery and the transcriptional regulation of the INK4/ARF locus [109–111]. The DDR machinery activates the p53-p21^CIP1^ axis, followed by inhibition of CDK2 activity, resulting in hyperphosphorylation of RB and cell cycle exit [109, 110, 112]. Moreover, DNA damage also induces the expression of *INK4*/*ARF* genes [113]. Under physiological conditions, the expression of *INK4-ARF* is arrested by epigenetic factors and polycomb proteins [114]. However, when INK4-ARF is activated, the increased level of ARF prevents p53 degradation and causes increased expression of p16^INK4a^*,* followed by inhibition of CDK4 and CDK6 and long-lasting cell cycle arrest [114]. In fact, DDR is a shared characteristic between cellular senescence and telomere shortening in aging [115]. Therefore, inefficient DNA repair under pathological conditions and aging results in cellular senescence and cell-cycle arrest [115].

#### Taupathy

Tau hyperphosphorylation is one of the typical pathologies in AD [116]. Tau is a tubulin-binding protein that promotes microtubule assembly and stabilization under physiological conditions [117]. The stabilization of microtubules is essential for fundamental metabolic and biochemical activities, including cell division, intracellular nutrient transport, cell motility, and cellular shape maintenance [66, 118]. However, under pathological conditions, tau hyperphosphorylation contributes to abnormal metabolic and biochemical activities in AD [66, 118, 119]. In AD, the abnormally phosphorylated tau dissociates from and destabilizes microtubules, and aggregates into oligomers, paired helical filaments, and intracellular neurofibrillary tangles [66, 118, 120]. There is increasing evidence for the close association between tauopathy and cellular senescence [121–123]. For example, a previous study found that the neurofibrillary tangle (NFT)-bearing neurons exhibited a senescence-associated transcriptomic profile in post-mortem AD brain tissues and tau-transgenic mice [122]. More interestingly, a senolytic therapy significantly ameliorated the NFT burden in the transgenic mice, suggesting the close association between hyperphosphorylated tau aggregation and cellular senescence [122]. Further, like neurons, astrocytes bearing pathological tau oligomers in human AD brains also display a senescence-like phenotype. Mechanistically, tau oligomers trigger the nucleo-cytoplasmic translocation of high mobility group box 1 (HMGB1) [121]. HMGB1 release causes the formation of SASP, which induces senescence in adjacent cells [121]. Treatment with HMGB1 inhibitors significantly abolished the tau oligomer-induced cellular senescence [121]. Furthermore, senescent microglia are closely associated with tauopathy and AD progression, although the underlying mechanisms remain unclear [124]. These findings suggest that tauopathy is involved in the cellular senescence in AD.

#### Aβ

Like hyperphosphorylated tau, oligomeric Aβ exposure induces cellular senescence in AD [125]. Aβ is generated from amyloid precursor protein (APP), a transmembrane protein [66]. APP is sequentially cleaved under physiological conditions by α- and γ-secretases, generating C-terminal fragments (p3, AICD, and CTF83) and soluble amyloid precursor protein α [66]. However, under pathological conditions, APP undergoes sequential cleavages by β- and γ-secretases to produce neurotoxic Aβ [66]. The excessive Aβ accumulation results in extracellular Aβ aggregation into amyloid plaques [66]. Numerous studies have found that Aβ accumulation triggers excessive activation of glial cells, neuroinflammation, oxidative stress, and mitochondrial dysfunction [66, 85, 126], all contributing to cellular senescence in AD [125]. For example, Aβ oligomers are able to induce senescence of OPCs and neurons through Aβ functional receptors and some proteins that regulate neuroinflammation and metabolic pathways [125, 127]. For instance, an in vitro study found that Aβ_1-42_ oligomers induce neural stem/progenitor cell senescence, as evidenced by significantly increased p16 and SA-*β*-gal [127]. During this process, formylpeptide receptor 2, one of the Aβ_1-42_ functional receptors, activates its downstream ROS-p38MAPK signaling, which limits neural stem/progenitor cell function and leads to failure of neurogenesis [127]. In another in vitro study, Aβ_1-42_ oligomers induced neuronal senescence by suppressing the sirtuin-1 level, while preservation of the sirtuin-1 level significantly attenuated Aβ-induced cell senescence [125]. These findings suggest that Aβ accumulation contributes to cellular senescence in AD.

#### Telomerase deficiency and telomere shortening

Telomerase, a ribonucleoprotein complex, is a telomere-terminal transferase necessary for maintaining telomere length by adding guanine-rich repetitive sequences to chromosome ends [128]. In AD, Aβ accumulation inhibits telomerase activity and causes telomere shortening [129]. Telomere shortening is one of the hallmarks of aging, wherein progressive telomere shortening eventually causes replicative senescence [130]. In aging and aging-related diseases including AD, telomerase deficiency and telomere shortening compromise neurogenesis and cause cellular senescence [15, 131]. In contrast, telomerase reverse transcriptase (TERT), a key component of telomerase, could preserve telomere length and alleviate or reverse brain tissue degeneration and cellular senescence in AD [132]. Interestingly, a previous study found that human neurons with TERT expression had no hyperphosphorylated tau detected, while neurons with tau pathology were not detected with TERT staining [133]. In addition, TERT-deficient animals show AD-like phenotypes and increased tau pathology [133, 134]. As tauopathy is closely associated with cellular senescence [121, 122], the telomerase deficiency may also contribute to cellular senescence by exacerbating tau pathology in AD [134]. Therefore, telomerase deficiency can cause both replicative senescence through telomere shortening and stress-induced premature senescence by exacerbating tau pathology, oxidative stress, and neuroinflammation.

### Cell types affected by senescence in AD

Cellular senescence, as an important player in the pathological progression of AD, has been identified in various cell types within AD brains [135]. In the following, we will introduce cellular senescence in the AD brain.

#### Astrocyte senescence in AD

Astrocytes are the most abundant glial cells in the central nervous system [136]. They play an essential role in maintaining the homeostasis of brain function, including synaptic support, axon guidance, and regulation of the blood–brain barrier (BBB) and blood flow [137–139]. As a critical regulator of inflammatory responses, astrocytes respond to pathological changes and exert anti-inflammatory effects at the early stage of AD. However, astrocytes are polarized into neurotoxic phenotypes at the late AD stage and exacerbate AD pathology [140].

Astrocytes undergo both replicative senescence and stress-induced senescence, with a series of senescent characteristics and markers, including growth arrest, increased p53 and p21 expression, and elevated SA-β-gal activity in vitro and in vivo [141, 142]. Studies in primary human and rodent astrocytes found that various stimuli and factors (such as H_2_O_2_, ionizing radiation, and proteasome inhibition) can lead to stress-induced senescence in astrocytes with classical senescence features [143, 144]. Senescent astrocytes display multiple classic senescent characteristics [143, 144]. First, senescent astrocytes exhibit cellular arrest both in vitro and in vivo [145]. As mentioned previously, the upregulation of p21^WAF1^ by p53 inhibits CDK2 activity and initiates cell cycle arrest [52]. Furthermore, downregulation or inhibition of CDK4 and CDK6 by p16^INK4a^ blocks the astrocytic entry to the S phase and mediates permanent cell cycle arrest [47]. Second, senescent astrocytes exhibit nuclear changes. A previous autopsy study found prominent typical chromatin structural changes (senescence-associated heterochromatic foci) and increased levels of histone modification (e.g., γH2AX) in astrocytes of AD patients [146]. The nuclear changes are associated with decreased expression of proliferation-related genes, leading to cell cycle arrest [145]. Third, astrocyte senescence is also characterized by the generation of SASP factors [147]. The SASP secretes pro-inflammatory factors, promotes the senescence state, and enhances age-related neurogenerative disease [144, 148].

The neuroprotective function of astrocytes is compromised in AD [149, 150]. Cellular senescence is one of the reasons that contribute to astrocyte dysfunction in AD [149, 150]. Senescent astrocytes lack normal function, acquire the SASP, and secrete inflammatory cytokines and proteases that affect the function of neighboring cells [13]. The population of p16^INK4a^-positive astrocytes is significantly increased in AD patients, suggesting prominent astrocyte senescence [13]. An in vitro study in human astrocyte cultures confirmed that Aβ_1-42_ peptides could elevate SA-β-gal activity and p16^INK4a^ expression, suggesting that excessive production of Aβ_1-42_ is involved in astrocytic senescent response [13]. In addition, the senescent astrocytes release multiple inflammatory cytokines, including IL-6, RANTES, IL-8, and ICAM-1, inducing a vicious circle [13]. Emerging evidence indicates that inhibiting astrocyte senescence or clearing senescent astrocytes could prevent AD initiation or slow AD progression [10, 151]. For example, a previous study using primary human astrocytes showed that inhibiting astrocyte senescence by administering the p53 isoform Δ133p53 may alleviate AD pathologies and confer neuroprotective effects [151]. Furthermore, eliminating senescent astrocytes via the genetic approach and pharmacological drugs (e.g., ABT263, dasatinib, and quercetin) alleviates Aβ plaque deposition, reduces neuroinflammation, and improves cognitive function in an ex vivo model of AD and in transgenic p16-3MR mice with cognitive deficits [152, 153]. These findings suggest that astrocyte senescence is a potential target for alleviating AD pathology.

#### Microglial senescence

Microglia, the primary immune cells in brain, play essential roles in neuroinflammation and brain infection [154, 155]. Moreover, they modulate synaptic pruning and eliminate unnecessary synapses in adulthood [156]. Excessive synaptic pruning mediated by adult brain microglia contributes to neurodegenerative diseases and related behavioral deficits [156]. Indeed, microglia exhibit multiple roles in AD initiation and development [67, 104, 154]. The increasing deposition of Aβ initiates the activation of microglia at early stages of AD to remove Aβ deposition through phagocytosis [34, 157]. However, with the progression of AD, especially at the late stage, the capability of microglia to clear amyloid plaques is compromised and they transform to a pro-inflammatory phenotype that contributes to AD progression [158]. Microglial activation is now understood to be a spectrum rather than a binary M1/M2 classification [159]. Microglia can be categorized into specific subsets such as disease-associated microglia (DAM), homeostatic microglia, intermediate states that exhibit characteristics of both pro-inflammatory and anti-inflammatory responses, and other subsets with disease-related genes and RNA signatures observed by single-cell RNA sequencing [155, 160–162]. At the early stage of AD, microglia predominantly exhibit a homeostatic or anti-inflammatory phenotype that facilitates Aβ clearance, while in the late stages, they transform to a disease-associated or pro-inflammatory state with diminished phagocytic capacity [162–167]. Regulation of microglial phagocytosis and microglial recruitment around amyloid plaques provides a potential approach for AD treatment [168, 169].

In addition, the DAM can secrete many same pro-inflammatory molecules as those found in the SASP, complicating the identification of senescent microglia [155, 160–167]. Several criteria can be considered to differentiate senescent microglia from DAM: (1) persistent inflammatory profile: senescent microglia maintain a chronic pro-inflammatory state, unlike the transient inflammatory responses seen in DAM [170]; (2) senescence markers: increased expression of senescence markers such as p16^INK4a^, p21^CIP1/WAF1^, and β-galactosidase activity can help identify senescent microglia [170]; (3) functional decline: senescent microglia often exhibit reduced phagocytic activity and impaired responses to injury or infection [170, 171]; (4) epigenetic changes: senescent cells may undergo specific epigenetic modifications, such as changes in DNA methylation and histone modification patterns, which can be applied as additional markers [172].

Senescent microglia have also been found in multiple brain regions in AD [154, 173]. In AD-like mouse models and post-mortem human brain samples, microglial senescence is detected in the cortex and hippocampus, contributing to Aβ pathology [154]. The generation of microglial senescence is correlated with sustained microglial proliferation at the early AD stage [154]. The replicative senescence is characterized by telomere shortening and elevated SA-β-gal activity, similar to other cellular senescence [154]. Specifically, senescent microglia are characterized by a decreased phagocytic ability, resulting in deficiency in Aβ clearance and Aβ accumulation in AD mouse models [154]. In addition, increased accumulation of extracellular Aβ and abnormal intracellular tau contribute to myelin loss at the early AD stage [34, 174]. Microglia are essential for phagocytosis and degradation of myelin debris [156]. However, when the myelin debris accumulation exceeds the degradation capacity of microglial lysosomes, microglia may become senescent and promote the release of pro-inflammatory cytokines [109]. The impaired lysosomal degradative capacity and the sustained secretion of inflammatory factors further lead to increased myelin debris and Aβ accumulation [109]. The excessive production of myelin debris and overload of myelin residues within microglia in turn lead to senescence. These changes disrupt microglial production of factors required for oligodendrocyte-dependent remyelination, suggesting that microglia senescence disrupts neuronal repair [109, 175].

Increasing evidence supports inhibiting cellular senescence as a new therapeutic paradigm for AD [154]. In an AD-like mouse model, inhibiting microglial proliferation at the early stage hinders microglial senescence and attenuates AD-associated pathology including Aβ accumulation and synaptic damage [154]. The *MAPT*^*P301S*^*PS19* transgenic mice exhibit a significant increase in the expression of senescence marker p16^INK4a^ at the early AD stage [10], preceding the initiation of aggressive tauopathy [10]. Eliminating the p16^INK4a^-expressing glial cells, including senescent microglia, prevents neurofibrillary tangle deposition and mitigates cognitive deficits [10].

#### OPC senescence

OPCs, also known as NG2-glia, oligodendrocyte precursor cells, or synantocytes [176], are the fourth major glial subtype in the brain and the source of oligodendrocytes [177]. Oligodendrocytes are the primary myelin-forming cells in the central nervous system (CNS) [176]. As the primary source of oligodendrocytes, OPCs are essential for myelination and myelin repair [176]. During the early development of brain tissue, OPCs migrate along the cerebral vasculature and spread to the entire brain [178]. The long-distance migration of OPCs supports the generation of oligodendrocytes and neuronal myelination [178]. OPC migration is guided by several signaling molecules, including Sonic hedgehog, bone morphogenic proteins, and Wnt proteins [179]. Similarly, several growth factors facilitate OPC migration, including hepatocyte growth factor, fibroblast growth factor, and vascular endothelial growth factor [179]. Once OPCs reach the specific brain region, the cells detach from the endothelium and differentiate into pre-oligodendrocytes. As oligodendrocytes mature, they contact neuronal axons and initiate myelin assembly [179, 180]. Indeed, the OPCs retain the ability to proliferate and differentiate throughout adulthood [181]. Following acute demyelinating injury or in demyelination-related neurodegenerative diseases, the OPCs migrate to the lesion area and differentiate into oligodendrocytes, contributing to myelin repair [182]. Dysfunction of OPCs contributes to demyelination and compromised remyelination in aging and neurodegenerative diseases [183]. The compromised remyelination with aging is characterized by poor response of OPCs to differentiation signals and weakened recruitment of OPCs to the damaged area [183]. Improving remyelination by rejuvenating OPCs has shown therapeutic potential for neurodegenerative diseases [183].

In AD, loss of OPCs occurs at an early stage and has been recognized as an early sign of AD pathology [176]. Elevated OPC senescence and functional disruption have been detected in the aging brain [176]. In addition, OPC senescence and demyelination were found to be aggravated in a transgenic AD mouse model [176]. Senescent OPC-related SASP contributes to local neuroinflammation and is closely associated with neuronal damage [7]. The SASP exacerbates neuronal damage and AD pathology by releasing pro-inflammatory factors [7]. The unfavorable inflammatory microenvironment in turn impairs neurogenesis and OPC differentiation and increases Aβ accumulation [176, 184, 185]. Myelin disruption is a typical change in AD and directly leads to memory deficits [174]. In 3 × Tg-AD mice, loss of myelin and OPCs is detected as early as 6 months of age, suggesting that the dysfunction of OPCs at the early stage of AD is not induced by replicative exhaustion but possibly by OPC senescence [176]. Indeed, OPCs around the senile plaques display the senescence phenotype in AD patients and AD animal models, suggesting that Aβ exposure causes OPC senescence [7].

Evidence from transgenic AD mice suggests that senolytic treatment targeting OPC senescence can alleviate typical AD pathological changes, including Aβ accumulation and hippocampal-dependent cognitive deficits [7]. Treatment with FDA-approved ‘senolytic’ compounds dasatinib and quercetin significantly alleviates pro-inflammatory cytokines, reducing Aβ accumulation in APP/PS1 AD mice [7]. In addition, treatments that promote OPC generation and differentiation exhibit promising therapeutic effects in AD [186, 187]. Preserving differentiation and reducing senescence of OPCs rescued short-term memory deficits and enhanced myelin formation in an AD mouse model [186]. As mentioned, the OPCs migrate along cerebral vasculature, spread to the entire brain, and contribute to remyelination [180]. Therefore, the loss of migration of senescent OPCs may contribute to perivascular clustering and impaired remyelination, although more studies are still needed [176, 188].

#### Neuronal senescence

Neurons are the fundamental units and the primary executor of signal transmission within the brain [189]. However, neuronal cells are often subjected to physiological and pathological stress, leading to elevated levels of DNA damage and an activated DDR [190]. The DNA damage causes aberrant cell-cycle re-entry for some neurons and leads to neuronal apoptosis [190]. However, the neuronal DDR promotes cell-cycle arrest and induces a senescence-like phenotype in neurons, which helps protect them from apoptotic death [190]. The surviving senescence-like neurons have all the features of cellular senescence, including mitochondrial dysfunction, metabolic dysfunction, and excessive release of pro-oxidant and pro-inflammatory cytokines [190].

The adult CNS neurons are often considered permanently postmitotic; however, this notion may not fully capture the complexities of their cell cycle regulation [191–193]. These highly specialized cells must consistently monitor their cell cycle status. Relaxation of this vigilance can trigger re-entry into the cell cycle, resulting in an altered and vulnerable state that often results in cell death [191–193]. Notably, neurons susceptible to neurodegeneration are also at an increased risk of initiating cell cycle processes characterized by expression of cell cycle proteins and DNA replication. This connection between cell cycle dynamics and neuronal death is fundamental to the pathology of various neurodegenerative diseases [191–193]. Depending on their maturation stage, neurons that do not adequately suppress cell cycle re-entry face different outcomes [191–193]. In younger, differentiating neurons, re-initiating the cell cycle can lead to cell death within hours, a process that can be prevented through cell cycle inhibition [191–193]. In contrast, adult neurons that re-enter the cell cycle may express cell cycle proteins and replicate portions of their genome. However, the cell cycle often remains prolonged, where they neither complete the cell cycle nor undergo cell death for an extended period [191–193].

Therefore, it is worth noting that defining neuronal senescence is a challenge in non-proliferating cells, such as post-mitotic and terminally differentiated neurons [194]. Historically, neurons were believed to escape from senescence, as senescence was thought to occur only in dividing cells. Consequently, most studies on brain cell senescence focused on glial cells [13, 154]. However, studies have demonstrated that post-mitotic neurons also undergo senescence, characterized by expression of markers like SA-β-gal, MCP-1, γ-H2AX, and 4-HNE, leading to the concept of amitosenescence [195, 196]. Long-term cultured neurons exhibit hallmark features of senescence earlier than glial cells, including increased SA-β-gal activity, SASP, DNA damage, and astrogliosis, which can be mitigated by senolytic agents such as resveratrol [197]. Neuronal senescence involves sustained DNA damage, high ROS production, altered transcription, increased p21^CIP1^ expression, and activation of the p38MAPK pathway [198]. Therefore, despite the challenges in defining senescence in neurons, a combination of alternative markers and functional changes may help determine whether neurons are truly senescent. These indicators include all the aforementioned cellular senescence markers.

Consistent with other cells within the brain [154], neuronal senescence is considered a source of oxidative stress and neuroinflammation, suggesting the potential contribution of neuronal senescence in aging and neurodegenerative diseases [198]. In a previous study, more than 97% of the senescent excitatory neurons overlapped with abnormal tau in AD post-mortem human brain tissue, suggesting the close association between neuronal senescence and typical AD pathology [199]. Consistent with these findings, in an AD transgenic mouse model, the expression of *Cdkn2a* was significantly increased in the NFT-containing neurons [122]. Furthermore, the cellular senescence markers, including p21, Cdkn2a, and p16^INK4a^, were colocalized with NFTs in the brain of a tau transgenic mouse model and elevated in human AD, suggesting the existence of neuronal senescence in both human and animal models [122]. Moreover, a study using the 5 × FAD mouse model further specified the cell types of senescence within the hippocampus [200]. Co-staining of p16 and other cellular markers confirmed that p16 was mainly located within neurons, and in a small proportion of astrocytes and microglia [200]. Notably, neuronal senescence occurs even at the early stage of AD, suggesting that neuronal senescence can be considered an indicator and a typical pathological characteristic of AD [200]. In vitro experiments confirmed the elevation of p16 expression and established the association between Aβ and neuronal senescence in cultured primary neurons [200].

Increasing evidence suggests that inhibiting neuronal senescence or clearing senescent neurons may be a possible treatment for AD [200]. In a tau-transgenic mouse model, clearance of senescent neurons significantly attenuated AD pathologies, including ventricular enlargement, neurofibrillary tangle burden, neuronal damage, and neuronal degeneration [200]. A recent study found that AD is characterized by elevated post-mitotic neuronal senescence in humans [194]. The senescent neurons release pro-inflammatory cytokines and cause a cascade of brain inflammation at the late AD stage [194]. However, reducing neuronal senescence to normal levels could ameliorate neuroinflammation and slow AD progression [194].

#### Endothelial cellular senescence

Brain endothelial cells are the major component of the microvasculature that forms the BBB, protecting the brain against toxins and pathogens and restricting the access of soluble and cellular substances from the blood into the brain [201]. As the main component of BBB, brain endothelial cells contribute to regulating local cerebral blood flow and neurovascular unit function [202, 203]. However, the brain endothelial cells undergo significant changes with age and experience senescence-associated stress during aging, including excessive oxidative stress, increased innate immunity, accumulation of DNA damage, and telomere shortening [204]. As an exquisite sensor of aging-associated circulatory cues, brain endothelial cells and BBB are potential therapeutic targets for AD [201, 205].

Increasing studies indicate that the senescence of endothelial cells contributes to the dysfunction of neurovascular units and BBB [204, 206]. Both in vivo and in vitro studies have demonstrated endothelial cell senescence induced by external stimuli and aging [207]. For example, γ-irradiation causes rat primary brain endothelial cells to acquire SASP, as evidenced by significantly increased DNA damage and upregulated expression of p16 and p53 [207]. These changes impair the angiogenic capacity [207]. In aged mice, significantly increased expression of senescence core genes, senescence effector genes, and SASP genes was detected in the brain, accompanied by markedly increased senescent endothelial cells [208]. The underlying mechanisms for endothelial cell senescence include oxidative stress, DNA damage, mitochondrial dysfunction, and inflammation [209, 210].

Mounting evidence suggests that endothelial cell senescence contributes to AD progression and pathogenesis [211]. A positive feedback relationship has been reported between NFT formation and cellular senescence [122]. In the prefrontal cortex of AD patients, expression of endothelial senescence-associated genes is elevated, contributing to AD-related BBB dysfunction and cerebral blood flow impairment [211]. Endothelial senescence has also been detected in the in vitro cell cultures [212]. In vitro studies showed that neurotoxic Aβ exposure induces endothelial cell senescence [213]. Although the causal relationship between endothelial cell senescence and AD remains unclear [109], DNA damage and senescence in the endothelial cells may lead to neurovascular unit dysfunction in elderly individuals with AD [214]. However, to the best of our knowledge, no studies have specifically investigated the effects of clearing senescent endothelial cells in AD.

## Targeting cellular senescence with senotherapeutics in AD

As mentioned previously, senescent cells acquire SASP in AD and other neurodegenerative diseases, releasing many inflammatory cytokines and matrix-degrading molecules [7, 215]. Therefore, accumulation of senescent cells induces chronic activation of the immune system, resulting in reduced senescent cell clearance and increased accumulation of senescent cells, thereby forming a vicious cycle [215]. Several potential therapeutic approaches that target senescent cells by clearing senescent cells or selectively blocking SASP using natural or synthetic compounds have been studied in animals and in clinical trials [7, 215]. These therapeutics include natural or synthetic compounds that are members of senolytics and senomorphics.

### Senolytics

Senolytics are small molecules that selectively clear senescent cells through cellular apoptosis [216]. Therefore, components that affect anti-apoptotic pathways may serve as senolytics. For example, Bcl-2 family inhibitors, BH3 mimetics, Hsp90 inhibitors, p53 binding inhibitors, HDAC inhibitors, p38MAPK inhibitors, and JAK/STAT inhibitors are widely used senolytics (Table 1) [215, 217–223]. Senolytics significantly alleviate AD pathology [7, 215]. For instance, dasatinib (a protein tyrosine kinase inhibitor) and quercetin (a natural compound that serves as a senomorphic) co-administration effectively removes senescent OPCs, alleviates neuroinflammation, attenuates Aβ accumulation, and improves cognitive function, suggesting the great potential of senescent cell clearance in AD therapy [7].

**Table 1:** Senotherapeutics targeting cellular senescence

Category	Drug name	Targets	References
*Senolytics*			
Bcl-2 family inhibitor/ BH3 mimetic	Navitoclax (ABT-263); ABT-737; A-1331852; A-1155463; Dasatinib + Quercetin (Phase 1 trial); Navitoclax (phase II clinical trial)	Bcl-2, BCL-X_L_, PI3K/Akt	[222, 223, 286]
Hsp90 inhibitor	17-DMAG (alvespimycin)	HSP90 (protein stabilization and degradation)	[221]
p53 binding inhibitor	PIK3R3; TRIAP1	P53/p21 signaling	[287, 288]
HDAC inhibitor	Bocodepsin (OKI-179)	Histone deacetylases	[289]
p38MAPK inhibitor	SB203580; UR13756; BIRB796	p38MAPK signaling	[217, 218]
JAK/STAT inhibitor	AG490; momelotinib; INCB18424	JAK/STAT	[219, 220]
*Senomorphics*			
PI3K/Akt/mTOR pathway inhibitor	Rapamycin (Phase II); Rapalogs (rapamycin analogs); Torin 1; NVP-BEZ235	mTOR	[290–293]
ATM/NF-κB signaling inhibitor	KU-60019; KU-55933	ATM	[268, 270]
Ca^2+^ channel inhibitor	Loperamide; NDGA; Isradipine	Ca^2+^ channel	[276, 294]
ERK pathway inhibitor	Simvastatin	ERK	[278]
MAPK	UR-13756; CDD-111; BIRB 796SB203580	MAPK	[272, 295, 296]
Receptor antagonist or inhibitors of the pro-inflammatory cytokine	Anakinra (IL-1 receptor); Tocilizumab/siltuximab (IL-6 receptor); IL-6 (sirukumab); Adalimumab/etanercept/infliximab (TNF-α inhibitor)	Inflammatory cytokines or pathway	[297–301]
JAK/STAT pathway inhibitor	Momelotinib; ruxolitinib; AG490; INCB18424	JAK1/2	[220, 302]
NF-κB inhibitor	NF-κB monoclonal antibodies	NF-κB	[226, 244]
p38MAPK inhibitor	p38MAPKα (shp38α) shRNA	p38MAPK	[218]
Metformin	Metformin (Phase III)	NF-κB nuclear translocation; IκB and IKKα/β phosphorylation; AMPK; mTOR; S6K	[244, 245, 254]
Non-steroidal anti-inflammatory drug	Aspirin	SIRT1; DNA damage	[109, 229]

In a tau-transgenic mouse model that displays late-stage pathology with a senescence-associated transcriptomic profile [122], dasatinib and quercetin (DQ) senolytic treatment for 12 weeks (two consecutive days every other week) significantly reduced NFT density, neuronal loss, and neurodegeneration, suggesting a strong association between cellular senescence and AD pathology [122]. Moreover, in APP/PS1 AD mice, Aβ plaque-associated OPCs display a senescence-like phenotype, suggesting that OPCs around the senile plaques lose their function in neuronal repair and demyelination [7]. Similarly, DQ senolytic treatment significantly alleviates AD pathology, as evidenced by attenuated Aβ accumulation and cognitive impairment [7]. Further mechanistic studies found that the DQ senolytic treatment ameliorates Aβ load by clearing senescent OPCs from the plaque environment and reducing SASP-induced excessive neuroinflammation, suggesting that senolytic treatment is a potential therapeutic approach for AD [7].

In addition to animal studies (Table 2), several senolytic therapies are currently undergoing clinical trials [224, 225]. In a phase I feasibility trial, oral QD administration was proven safe, with dasatinib showing a good profile of BBB penetration [224]. Although cognitive and neuroimaging endpoints did not show improvement after treatment, CSF levels of senescence-related cytokines and chemokines displayed a trend of decrease and CSF Aβ levels showed a trend of increase. These findings suggest the potential of QD in treating AD under safety, tolerability, and feasibility [224].

**Table 2:** Recent animal studies targeting senescent cells in AD

Animal model	Methods/drugs targeting senescent cells	Outcomes	Targeted cells	Ref
APP/PS1 AD mice	Dasatinib and quercetin	Attenuated Aβ accumulation and cognitive impairment	OPCs	[7]
*MAPT*^*P301S*^*PS19* mice	ABT263 (navitoclax); genetic clearance	Prevented gliosis, neurofibrillary tangle deposition, and neurodegeneration, and preserved cognitive function	Microglia and astrocytes	[10]
APP/PS1 model	CSF1R inhibitor (GW2580)	Alleviated Aβ accumulation as well as neuritic and synaptic damage	Microglia	[154]
Aged rats	Dasatinib and quercetin	Alleviated age-associated cognitive deficits and peripheral inflammation, and preserved synaptic plasticity	No specific cells mentioned	[303]
Aged mice	Geneticic clearance with AP20187; dasatinib and quercetin	Improved cognitive function	Whole-body senescent cell clearance	[304]
Tau transgenic mice	Dasatinib and quercetin	Alleviated NFT density, neuron loss, and ventricular enlargement; improved aberrant cerebral blood flow	No specific cells mentioned	[122]

### Senomorphics

Senomorphics are molecules that inhibit or block some of the characteristics of SASP without killing senescent cells [215]. Currently, therapies using senomorphics adopt two major therapeutic strategies [215], targeting the SASP-associated pathways (PI3k/Akt, JAK/STAT, and mTOR pathways, etc.) and transcription factors (NF-κB, C/EBP β, and STAT3 factors, etc.) [226], and neutralizing the SASP factors, including inflammatory cytokines and matrix-degrading molecules [226]. For example, monoclonal antibodies against IL-6, NF-κB, and IL-8 effectively alleviate SASP-induced chronic inflammation [226, 227]. However, senomorphics display apparent defects [215]. For example, senomorphics should be administered chronically to achieve sufficient effectiveness [215]. However, long-term senomorphics administration may inhibit pathways and transcription factors essential for maintaining tissue homeostasis [215]. According to previous studies, the most widely studied senomorphics suppress SASP by targeting NF-κB, IL-1α, mTOR, JAK/STAT, and p38MAPK pathways [228, 229].

#### Rapamycin

Rapamycin isolated from the bacterium *Streptomyces hygroscopic*us is one of the most widely used senomorphics [230]. Rapamycin was initially developed as an anti-fungal agent and later applied to prevent organ rejection and treat lymphangioleiomyomatosis [231]. Currently, rapamycin is one of the most widely established senomorphics that alleviate cellular senescence and the characteristics of SASP [229]. Rapamycin reduces cellular senescence mainly by inhibiting the mTOR signaling [232]. The mTOR contains two main functional complexes: mTORC1 and mTORC2 [233]. Increased mTORC1 activity has been found to cause phenotypes of cellular senescence, and inhibiting mTORC1 activity is currently one of the best-known pharmacological approaches to attenuate cellular senescence and increase lifespan [234, 235]. As an acute inhibitor of mTORC1, rapamycin inhibits translation and cell growth by inhibiting the phosphorylation of downstream substrates, including eukaryotic translation initiation factor 4E-binding protein 1 (4E-BP1) and S6 kinase 1 (S6K1) [236]. 4E-BP1 and S6K1 are involved in cell growth, proliferation, and migration by regulating mRNA translation and protein synthesis [237, 238]. Although many studies have demonstrated beneficial effects of rapamycin in alleviating AD pathology, including reducing Aβ accumulation [239], tau hyperphosphorylation [240] and neuroinflammation [241], and improving cognitive dysfunction [242], the effects of rapamycin remain controversial. For example, a previous study found that treating AD mice with rapamycin causes reduction of Aβ clearance [243]. Therefore, more studies investigating the effects of rapamycin on AD are warranted.

#### Metformin

Metformin is another widely studied senomorphics, which was initially developed for treating type 2 diabetes [229]. Metformin alleviates cellular senescence and SASP by inhibiting nuclear translocation of NF-κB and preventing IκB and IKKα/β phosphorylation, which is AMPK-independent [244]. However, another study found that metformin attenuates stress-induced cellular senescence and restores almost all senescence-related functions in an AMPK-dependent manner [245]. This discrepancy may be caused by differences in the types of cell and senescence. Increasing studies have attempted to repurpose metformin to AD therapy [246–248]. Clinical evidence suggests that metformin could reduce the risk of AD in elderly diabetic patients and exert beneficial effects in patients with cognitive impairment [249–251]. Moreover, animal studies showed that metformin could alleviate typical AD pathology, including tau pathology, Aβ pathology, neuronal loss and dysfunction, and neuroinflammation [252–255]. Mechanistically, metformin markedly promotes the expression of neurotrophic factors (e.g., BDNF and NgF), which exert neuroprotective effects and significantly improve synaptic plasticity [256, 257]. Moreover, metformin could attenuate Aβ deposition in AD by increasing insulin-degrading enzyme (an Aβ-degrading peptidase) levels [258]. Metformin also decreases Aβ production by upregulating AMPK expression and suppressing the activation of mTOR, P65 NF-κB, and S6K [254]. As mentioned previously, the activation of mTOR, NF-κB, and S6K is a typical change in cellular senescence. Therefore, the effects of metformin on mTOR, NF-κB, and S6K in AD indicate that metformin could alleviate cellular senescence in AD [254]. The decreased pro-inflammatory cytokines following metformin treatment further support the attenuation of SASP in AD [254]. These findings suggest that metformin, as a widely studied senomorphics, holds a promising therapeutic potential in alleviating cellular senescence and AD pathology.

#### NF-κB inhibitors

NF-κB is one of the main inducers of SASP [259]. The mammalian NF-κB family has five members: RelA (p65), RelB, c-Rel, NF-κB1 (p50/p105), and NF-κB2 (p52/p100) [260]. There are two types of NF-κB signaling pathways, canonical and noncanonical. Activation of p65 homodimers or c-Rel/p65 heterodimer causes the activation of the canonical NF-κB pathway in response to pro-inflammatory cytokines and bacterial products [260]. The noncanonical pathway is activated in response to stimuli involved in lymphoid organogenesis and causes activation of the p52/RelB heterodimer [261]. According to previous studies, NF-κB plays an indispensable role in cellular senescence [262, 263]. For instance, chronic activation of the canonical IKK/NF-κB signaling pathway and p65 phosphorylation increases the expression of neuroinflammatory markers and the senescence of oligodendrocytes, leading to white matter loss and neurological deficits [262]. Several signaling pathways are involved in the activation of NF-κB signaling and the induction of SASP, including DNA damage, p38MAPK signaling pathway, RIG-1 signaling pathway, TGF-β-TAK1 pathway, HMGB1 proteins, and ceramide signaling [259, 264]. Ataxia-telangiectasia mutated (ATM) kinase, a critical protein involved in DDR and DNA repair, causes activation of NF-κB and induces NF-κB-dependent, DNA damage-induced senescence [264]. Therefore, NF-κB inhibitors are potential senomorphic agents to inhibit SASP [226, 244]. Monoclonal antibodies or metformin can inhibit NF-κB and alleviate SASP-induced chronic inflammation [226].

#### ATM inhibitors

ATM, an essential protein involved in DNA damage and repair, promotes cellular senescence. ATM inhibitors can counteract cellular senescence [264]. In addition to inducing cellular senescence by activating NF-κB, ATM also disrupts the removal of defective mitochondria by autophagy [265, 266]. The accumulation of defective mitochondria induces inflammatory response and metabolic dysfunction, exacerbating cellular senescence [265, 267]. In contrast, ATM inhibition preserves mitochondrial function and promotes metabolic reprogramming, which is essential in alleviating cellular senescence during aging [268]. In a previous study, ATM depletion attenuates SASP in cellular senescence by reducing secretion of a large subset of major pro-inflammatory cytokines, including IL-6 and IL-8 [269]. These findings suggest that ATM inhibitors are promising strategies against SASP in aging and aging-associated neurodegenerative disorders [269]. KU-55933 and KU-60019 are the most widely studied ATM inhibitors [268, 270], which attenuate senescent markers and alleviate SASP [264, 268].

#### p38MAPK inhibitors

p38MAPK is a member of the mitogen-activated protein kinase (MAPK) family, and can be rapidly activated by phosphorylation in response to DNA damage, pro-inflammatory cytokines, and other extracellular stress [271]. p38MAPK activation is essential in stress-induced cellular senescence [218, 272]. For example, X-irradiation-induced genotoxic stress significantly increases p38MAPK phosphorylation and cellular senescence, wherein p38MAPK activation is necessary for SASP and the secretion of some SASP components [218]. Constitutive p38MAPK activation is sufficient to induce arrest of cell proliferation, increased expression of cellular senescence markers (e.g., SA-β gal activity), and SASP [218]. In contrast, p38MAPK inhibition alleviates cellular senescence and mitigates SASP component levels [218]. Moreover, further analysis revealed that the role of p38MAPK in cellular senescence and SASP is primarily achieved by increasing NF‐κB transcriptional activity [218]. Notably, although p38MAPK activation is involved in DNA damage-induced cellular senescence, p38MAPK activation induces SASP independently of the DDR signaling [218].

#### Other senomorphics

As mentioned, monoclonal antibodies against SASP-released inflammatory cytokines could alleviate SASP by neutralizing the released pro-inflammatory cytokines [226, 227]. Likewise, the non-steroidal anti-inflammatory drug Aspirin effectively reduces cellular senescence [125, 273]. For example, Aspirin could alleviate Aβ-induced cell senescence and DNA damage in human neurons and neural stem cells by rescuing SIRT1, suggesting Aspirin as a potential senomorphic drug in alleviating cellular senescence in AD [125, 274]. Moreover, rescuing SIRT1 has been shown to rescue Aβ-induced neuronal senescence and alleviate senescence-associated DNA damage. Additionally, the JAK-STAT signaling pathway, which is involved in the production of inflammatory cytokines, plays a critical role in cellular senescence and SASP [219, 220, 275]. Consistently, numerous studies confirmed that JAK/STAT inhibitors, including AG490, momelotinib, and INCB18424, could alleviate cellular senescence and SASP [219, 220]. In addition, senescent cells display increased mitochondrial and cytosolic Ca^2+^ levels, and Ca^2+^ channel inhibitors alleviate the intracellular Ca^2+^ rise-induced exacerbation of cellular senescence [276, 277]. Furthermore, there is additional evidence suggesting that the ERK pathway inhibitor simvastatin can suppress cellular senescence, although more evidence is needed to clarify its role in cellular senescence within the CNS [278].

## Conclusion

**Fig. 2:**
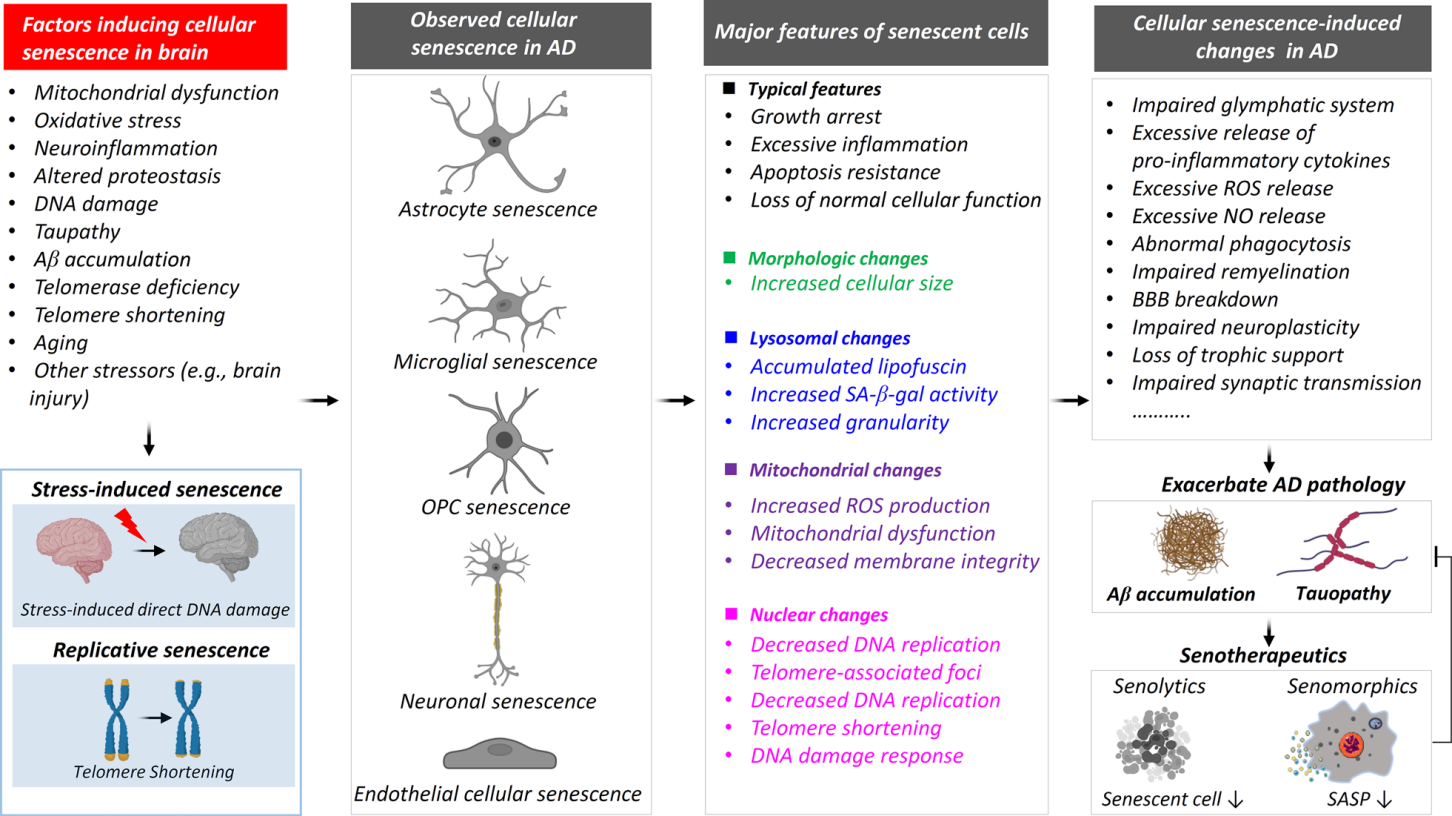
Summary of cellular senescence in AD. Cellular senescence, including stress-induced senescence and replicative senescence in the brain, is induced by multiple factors. Moreover, the factors inducing cellular senescence affect various cell types in AD, including astrocytes, microglia, oligodendrocyte precursor cells (OPCs), neurons, and endothelial cells. The senescent cells further exacerbate AD pathology. Senotherapeutics, which include senolytics and senomorphics, are strategies to alleviate cellular senescence. Senolytics promote the apoptosis of senescent cells, clearing them from the system, while senomorphics mitigate the SASP (senescence-associated secretory phenotype), thereby attenuating cellular senescence. Clearing cellular senescence holds promise for alleviating AD pathology

Cellular senescence in the brain is influenced by multiple factors, including mitochondrial dysfunction, oxidative stress, neuroinflammation, altered proteostasis, DNA damage, taupathy, Aβ accumulation, telomerase deficiency, telomere shortening, aging, and other stressors such as brain injury **(**Fig. 2). These factors lead to stress-induced senescence and replicative senescence. Moreover, the factors inducing cellular senescence contribute to cellular aging in AD, affecting various cell types including astrocytes, microglia, OPCs, neurons, and endothelial cells [145, 173, 176, 200]. With the progression of AD, the accumulated senescent cells acquire SASP and secrete numerous chemokines and pro-inflammatory cytokines, including pro-inflammatory factors, ROS, and matrix-degrading enzymes, to exacerbate AD pathology [279]. In addition, the loss of normal functions of the senescent cells in the brain tissue induces synaptic dysfunction, impaired remyelination, BBB breakdown, impaired remyelination, and impaired removal of neuronal debris, metabolic waste, and cell fragments [13, 109, 280, 281] (Fig. 2). Several strategies have been proposed to alleviate cellular senescence or SASP, including promoting apoptosis of senescent cells [7, 282], inhibiting oxidative stress [283], suppressing the cellular senescent-associated pathways [218, 268], destroying senescent cells using oncolytic viruses [284], or using monoclonal antibodies against SASP-released cytokines [226, 227]. Targeting cellular senescence with senotherapeutics has emerged as a promising approach to mitigating AD progression [125, 225, 274]. Senolytic drugs that selectively eliminate senescent cells, and senomorphic agents that suppress SASP without inducing cell death, are being investigated for their potential to alleviate AD pathology and improve cognitive function [216, 285]. Although increasing preclinical studies have demonstrated the efficacy of senotherapeutics in ameliorating AD-related phenotypes in animal models [7, 215], the safety and effectiveness of senotherapeutic interventions in human patients remain unclear. In addition, while this review offers an in-depth examination of these topics, we also acknowledge the limitations and gaps that remain in the current literature. For instance, not all instances of cellular senescence may occur simultaneously across cell types, and there is currently no direct evidence linking cellular senescence to the pathogenic mechanisms of AD. Further research is still needed to elucidate the specific mechanisms by which cellular senescence contributes to AD pathogenesis, and to optimize the safety and efficacy of senotherapeutic interventions in AD patients.

Moreover, it should be noted that while cellular senescence is associated with various pathological pathways and brain cell types in AD, the impact of senescence is not uniform across these cell types. Senescent astrocytes, microglia, neurons, OPCs, and endothelial cells exhibit distinct roles in AD pathology, contributing to disease progression through unique mechanisms. This highlights the complexity of senescence-related changes in AD and underscores the need for targeted approaches considering the diverse roles of senescent cells across the brain. In addition, as mentioned previously, aging is a critical state that increases the risk of developing AD. Cellular senescence associated with aging can lead to cumulative effects on brain health, contributing to neurodegenerative processes. The age-related senescent changes can exacerbate AD pathology by impairing neuronal function, increasing neuroinflammation, and disrupting homeostasis in the brain. Therefore, it is crucial to consider the interplay between aging and cellular senescence in the context of AD to fully understand its pathogenesis and identify potential therapeutic targets.

Finally, the relationship between cellular senescence and AD is complex, as the observed changes in senescent cells may be both causes and effects of AD pathogenesis. This bidirectional relationship complicates our understanding of the underlying mechanisms. Therapeutic strategies targeting cellular senescence in AD are still under investigation. Future research is essential to elucidate the causal relationships between cellular senescence and AD, which may provide insights into more effective therapeutic approaches.

### Limitations

While this review discusses the potential role of cellular senescence in AD pathology, it is important to acknowledge several limitations. First, there is currently a lack of experimental evidence demonstrating a clear causal relationship between cellular senescence and AD progression in human patients. Second, this narrative review does not follow a systematic review methodology, and no formal search strategy was employed. This may introduce selection bias in the sources cited and potentially limit the comprehensiveness of the literature reviewed. It is also important to note that while this narrative review focuses primarily on cellular senescence and apoptosis, other mechanisms, such as aberrant re-entry into the cell cycle, may also play a significant role in AD pathology. Finally, this review highlights several key factors that contribute to cellular senescence, such as oxidative stress, mitochondrial dysfunction, and telomere shortening; however, the relative weight and interactions of these factors in different cell types in the context of AD remain unclear. Further research is required to delineate these complex interactions and how they vary across different stages of AD.

## Data Availability

Not applicable.

## Abbreviations

AD: Alzheimer’s disease
OPC: Oligodendrocyte precursor cell
SASP: Senescence-associated secretory phenotype
Aβ: Beta-amyloid
SIPS: Stress-induced premature senescence
SA-β-gal: Senescence-associated beta-galactosidase
NSC: Neural stem cell
CNS: Central nervous system
NFT: Neurofibrillary tangle
BBB: Blood-brain barrier
ROS: Reactive oxygen species
NAD +: Nicotinamide adenine dinucleotide
mTORC1: Rapamycin complex 1
mTOR: Mammalian target of rapamycin
DDR: DNA damage response
CDK2: Cyclin-dependent kinase 2
Rb: Retinoblastoma
HMGB1: High mobility group box 1
APP: Amyloid precursor protein
TERT: Telomerase reverse transcriptase
DQ: Dasatinib and quercetin
MAPK: Mitogen-activated protein kinase
